# Characterization of Conserved and Novel microRNAs in *Lilium lancifolium* Thunb. by High-Throughput Sequencing

**DOI:** 10.1038/s41598-018-21193-4

**Published:** 2018-02-13

**Authors:** Xiangfeng He, Awraris Getachew Shenkute, Wenhe Wang, Shufa Xu

**Affiliations:** 1 College of Landscape Architecture, Beijing University of Agriculture, Beijing, 102206 China; 2 Beijing Collaborative Innovation Center for Eco-Environmental Improvement with Forestry and Fruit Trees, Beijing, 102206 China; 3 Beijing Laboratory of Urban and Rural Ecological Environment, Beijing, 100083 China; 4 Key Laboratory of Pollinating Insect Biology, Ministry of Agriculture, Institute of Apicultural Research, Chinese Academy of Agricultural Sciences, Beijing, 100093 China

## Abstract

MicroRNAs (miRNAs) are among the class of noncoding small RNA molecules and play a crucial role in post-transcriptional regulation in plants. Although *Lilium* is one of the most popular ornamental flowers worldwide, however, there is no report on miRNAs identification. In the present study, therefore, miRNAs and their targets were identified from flower, leaf, bulblet and bulb of *Lilium lancifolium* Thunb. by high-throughput sequencing and bioinformatics analysis. In this study, a total of 38 conserved miRNAs belonging to 17 miRNA families and 44 novel miRNAs were identified. In total, 366 target genes for conserved miRNAs and 415 target genes for novel miRNAs were predicted. The majority of the target genes for conserved miRNAs were transcriptional factors and novel miRNAs targeted mainly protein coding genes. A total of 53 cleavage sites belonging to 6 conserved miRNAs families and 14 novel miRNAs were identified using degradome sequencing. Twenty-three miRNAs were randomly selected, then, their credibility was confirmed using northern blot or stem-loop qRT-PCR. The results from qRT-PCR analysis showed the expression pattern of 4 LL-miRNAs was opposite to their targets. Therefore, our finding provides an important basis to understand the biological functions of miRNAs in *Lilium*.

## Introduction

MicroRNAs (miRNAs) are a class of 20–24 nucleotide (nt) noncoding small RNA molecules and play a crucial role in post-transcriptional regulation in animals and plants^1,2^. In plants, microRNA genes are transcribed by RNA polymerase II into primary miRNAs (pri-miRNAs) with a cap and a poly(A) tail. The pri-miRNAs are then processed into hairpin precursors (pre-miRNAs) by a protein complex consisting of the Dicer-like 1 (DCL1), the C2H2-zinc finger protein SERRATE 11(SE), and the double-stranded RNA-binding protein HYPONASTICLEAVES1 (HYL1)^3^. The miRNA duplexes (miRNA/miRNA*) are released from pre-miRNAs by DCL1 and each strand in the miRNA duplex is methylated^3^. The miRNA strand is loaded into the ARGONAUTE (AGO) protein of RNA-induced silencing complex (RISC) to carry out its function^1,3^. Several research evidences revealed that miRNAs play important roles in diverse biological processes including plant growth, development, biotic and abiotic stress responses, and signal transduction^4–6^.

The first miRNA, lin-4, was identified from *Caenorhabditis elegans* in 1993^7^. In plant, the first miRNAs were identified from *Arabidopsis*^8^. Following that, some miRNAs have been identified from plants using cloning or bioinformatics prediction^9–12^. The high-throughput sequencing technology was firstly used to identify *A.thaliana* miRNAs in 2005^13^. Since then, thousands of miRNAs from different species have been discovered by high-throughput sequencing technology^14–19^. To date, a total of 35,828 mature miRNAs sequences from 223 different species (ranging from viruses to humans) have been identified according to the miRBase database (release 21, June 2014). However, there are only few researches conducted on miRNAs identification in ornamental flowers, including *Phalaenopsis aphrodite*, *Rosa hybrida*, *Aquilegia coerulea* and *lotus japonicas*^20–23^.

*Lilium* is a genus of Liliaceous perennial bulb plants. Lily (*Lilium* spp.), owing to their large and colorful flowers, have become one of the most popular ornamental flowers worldwide^24^. In addition to their ornamental value, some lily species are edible and has long been used as traditional medicine in China and Korea^25–28^. The genus *Lilium* has more than 100 species worldwide, of which 55 species and 18 varieties are originated in China^29^. *Lilium lancifolium* Thunb. is a well-known lily species widely distributed in China, and is often planted for landscaping design. It is one of *Lilium* species formed Asiatic hybrid lilies by interspecific crosses and give them the characteristics of orange flower and raised spot in petal^30^. Several studies have revealed its better capacity for resisting high and low temperature, drought, disease and changing soil salinity than other lilies^31,32^. Therefore, it has been used to produce progeny with desirable stress resistance in lily hybrid breeding^32^. Although several research reports shown that miRNAs play crucial roles in plant growth, development and response to stress in plant, to the best of our knowledge there is currently no report on *Lilium* miRNAs. In this study, therefore, we employed the high-throughput sequencing and bioinformatics analysis to identify miRNAs and their targets in *L. lancifolium*.

## Results

### Construction and sequencing of small RNA libraries

**Figure 1:**
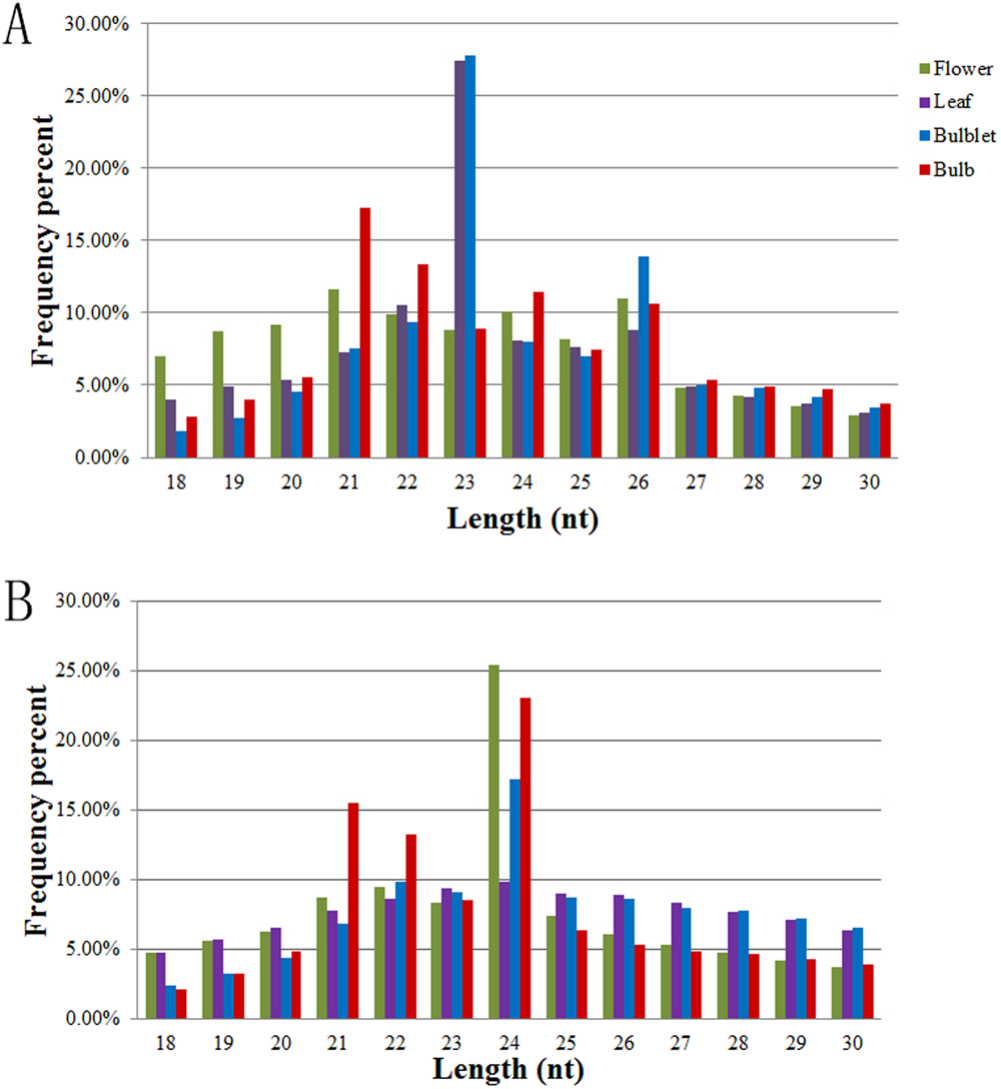
The length distribution of the clean and unique reads from flower, leaf, bulblet and bulb of *L. lancifolium*. (**A**) Clean reads; (**B**) Unique reads.

To identify the miRNAs in *Lilium*, total RNAs were extracted from flower, leaf, bulblet and bulb of *L. lancifolium* and then used to construct four small RNA libraries (Supplementary Table S1). Then the four small RNA libraries were sequenced using Illumina HiSeq. 2500 sequencing platform and analyzed in bioinformatics. A total of 19,025,905 raw reads from flower, 19,636,648 from leaf, 22,776,684 from bulblet, and 18,676,061 from bulb were obtained. After removing adaptors, low quality reads and contaminants, 14,090,897clean reads from flower, 15,279,574 from leaf, 17,560,878 from bulblet and 14,935,394 from bulb were obtained (Table 1). The clean reads and unique reads of four tissues were subjected to analysis of the size distribution as shown in Fig. 1. The majority of the clean reads of small RNAs in four samples were 21 to 26 nt in size. The 21 nt class was the most abundant in flower and bulb, followed by 22, 24 and 26 nt classes (Fig. 1A). In leaf and bulblet 23 nt small RNAs are the most frequent, followed by 26, 22 and 24 nt. However, the 24 nt peak is found to be dominant at a unique read level in all four samples (Fig. 1B).

**Table 1:** Statistics of sequencing reads from flower, leaf, bulblet and bulb libraries of *L. lancifolium*.

Samples	Raw reads	Clean reads (18–30 nt)	Unique reads	Mapped reads	rRNA/tRNA/ snRNA/snoRNA	Without annotation
Flower	19,025,905	14,090,897	1,710,130	9,552,373	8,453,061	5,634,868
Leaf	19,636,648	15,279,574	1,103,780	10,493,835	10,263,031	5,012,994
Bulblet	22,776,684	17,560,878	1,394,751	12,803,064	11,776,773	5,764,221
Bulb	18,676,061	14,935,394	1,965,587	88,931,80	7,156,402	7,774,593

### Conserved miRNAs in *L. lancifolium*

To analyze the population of conserved miRNAs in *L. lancifolium*, sRNA sequences of the four libraries were compared with known mature miRNAs from other plants and *Lilium* RNA sequence. A total of 38 conserved miRNAs precursors were identified from four sRNA datasets, as shown in Table 2 and Supplementary Table S2. All these conserved miRNAs belong to 17 miRNA families and in most miRNA families, more than one precursors were identified. Among them, MIR156 and MIR159 were the largest families identified with five members, followed by MIR166, in which four members were identified. However, several miRNA families possessed only one precursor, including MIR162, MIR167, MIR172, MIR390, MIR399, MIR408 and MIR845. In most miRNA families, at least one conserved miRNA precursor with miRNA* from small RNA sequencing was identified (Supplementary Fig. S1).

**Table 2:** Conserved miRNAs identified from flower, leaf, bulblet and bulb libraries of *L. lancifolium*.

Family	miRNA	Number of precursor sequence	Sequence (5′-3′)	Length (nt)	Clean reads			
					Bulblet	Bulb	Flower	Leaf
MIR156	LL-miR156a	c45184.graph_c0	ugacagaagagagugagcac	20	263	29	32	2
		c51027_g1						
		c103364_g2						
		c205656_g1						
	LL-miR156i	c45184.graph_c0	ugacagaaagaguagugagca	21	8	1	2	1
MIR159	LL-miR159a	CL2574.Contig1_All	uggauugaagggagcucuaca	21	11	13	2	4
		CL2574.Contig4_All						
	LL-miR159b	c117583_g1	uuuggauugaagggagcucua	21	1446	969	671	379
		c44060.graph_c0						
	LL-miR319a	c120927_g2	uuggacugaagggagcucccu	21	1	2	0	0
MIR160	LL-miR160a	c19797.graph_c0	ugccuggcucccuguaugcca	21	3	1	8	3
		c50045_g1						
	LL-miR160f	c99102_g1	cugccuggcucccugaaugcc	21	1	18	2	3
MIR162	LL-miR162a	Unigene26825_All	ucgauaaaccucugcauccgg	21	17	52	50	4
MIR164	LL-miR164a	c96196_g1	uggagaagcagggcacgugca	21	11	0	18	0
		c30311.graph_c0						
MIR166	LL-miR166f	Unigene32510_All	ucucggaccaggcuucauucc	21	49	40	366	6
		c39808.graph_c0						
		c121414_g2						
	LL-miR166g	c106540_g1	ucggaccaggcuucauuccuc	21	369	122	259	92
MIR167	LL-miR167a	c105919_g1	ugaagcugccagcaugaucuga	21	7	37	1923	132
MIR168	LL-miR168a	c71740.graph_c0	ucgcuuggugcaggucgggaa	21	40	137	6	4
		c24174.graph_c0						
		c105404_g1						
MIR172	LL-miR172a	c79803.graph_c0	agaaucuugaugaugcugcaa	21	0	0	24	0
MIR390	LL-miR390a	c7928.graph_c0	aagcucaggagggauagcgcc	21	4	0	16	0
MIR395	LL-miR395a	c12817.graph_c0	ugaaguguuugggggaacucc	21	208	1183	105	140
	LL-miR395k	c5100.graph_c0	ugaagcguuugggggaacucc	21	0	2	1	1
MIR396	LL-miR396a	c103053_g1	uuccacagcuuucuugaacug	21	33	123	19	15
		Unigene25901_All						
	LL-miR396f	c30849_g1	uuccacggcuuucuugaacua	21	38	78	81	5
MIR398	LL-miR398b	c221661_g1	uguguucucaggucaccccug	21	55	70	11	71
		c35683_g1						
MIR399	LL-miR399a	c191701_g1	ugccaaaggagacuugcccug	21	2	0	3	5
MIR408	LL-miR408b	c121837_g2	ugcacugccucuucccuggcu	21	7	0	2	14
MIR845	LL-miR845	c54746.graph_c0	cgcucugauaccacuuguugg	21	1	8	1	0
MIR2118	LL-miR2118e	c28582_g1	uucccaaugccucucaugccaa	22	2	0	10	1
	LL-miR2118a	Unigene6944_All	uugccgauaccacccauaccga	22	4	6	3	0

The sRNA sequencing results indicated that the clean reads of conserved miRNAs ranged from 1 up to more than 1,000 in 4 samples. Of all conserved miRNAs, the clean reads of MIR159, MIR167 and MIR395 exceeded 1,000 in one tissue. The clean reads of four miRNA families (MIR156, MIR166, MIR168 and MIR396) ranged from 100 to 1,000 at least in one tissue. However, the other miRNA families (MIR160, MIR162, MIR164, MIR172, MIR390, MIR398, MIR399, MIR408, MIR845 and MIR2118) had fewer than 100 reads in all four tissues (Table 2).

In a broader evolutionary context, *L. lancifolium* miRNAs were aligned to those of 13 other plants, including 11 monocotyledons (*Aegilops tauschii*, *Brachypodium distachyon*, *Elaeis guineensis*, *Festuca arundinacea*, *Hordeum vulgare*, *Oryza sativa*, *Sorghum bicolor*, *Saccharum officinarum*, *Saccharum* sp., *Triticum aestivum* and *Zea mays*) and 2 dicotyledons (*Arabidopsis thaliana* and *Glycine max*). Of the 17 *L. lancifolium* miRNA families, 15 were conserved in more than 6 plant species. These miRNAs were considered as well-conserved miRNA families. However, LL-miR845 and LL-miR2118 from *L. lancifolium* were found in only two and one plant species, respectively (Supplementary Table S2).

### Novel miRNAs in *L. lancifolium*

To identify novel miRNAs that may be specific to *L. lancifolium*, all unannotated sRNAs were searched against the unigenes from *Lilium* transcriptome sequencing and EST from NCBI database using miRDeep2. After searching for potential precursors (pre-miRNAs) and predicting their stem-loop hairpin secondary structures, a total of 44 novel miRNAs were identified in four libraries. The novel miRNA sequences ranged in length from 20–24 nt. However, the sequences of most novel miRNAs were 21 nt length and started with a 5′-U (Table 3). The pre-miRNAs ranged in length from 127–1475 nt. The average minimum folding free energy value of the hairpin structures was −152 kcal/mol in *L. lancifolium* (Supplementary Table S3), which is higher than −76.8 kcal/mol found in *Arabidopsis*^33^. The structures of 44 novel miRNA precursors are shown in Supplementary Fig. S2. Nineteen miRNA* sequences from small RNA sequencing were discovered in these novel miRNA precursors. Half of novel miRNAs had more than 10 clean reads in at least one tissue. Only LL-miR09, LL-miR23 and LL-miR25 had more than 100 clean reads in leaf, bulb or flower tissue (Table 3). It is very interesting that two highly similar novel miRNAs, LL-miR07 and LL-miR14, were identified. Only two different bases were found between LL-miR07 and LL-miR14 mature miRNA sequences (Table 3). Moreover, the similarity of precursors between LL-miR07 and LL-miR14 was more than 85%.

**Table 3:** Novel miRNAs identified from flower, leaf, bulblet and bulb libraries of *L. lancifolium*.

miRNA	Number of precursor sequence	Sequence (5′-3′)	Length (nt)	Clean reads			
				Bulblet	Bulb	Flower	Leaf
LL-miR01	c33038.graph_c0	uaguaaguuugcagagcagag	21	0	0	53	0
LL-miR02	c1069.graph_c1	cuugugcuucuggacugcucc	21	0	56	0	0
LL-miR03	c15590.graph_c0	aagguauagagucagacacuu	20	0	0	9	0
LL-miR04	c25608.graph_c0	agacgaucgcaccaaacuggcuau	24	13	67	1	0
LL-miR05	c28639.graph_c0	uuuucuaugucacucaauccaa	22	0	2	3	0
LL-miR06	c30876.graph_c0	aguaaguugagaagaguaggagaa	24	3	3	0	1
LL-miR07	c31471.graph_c0	uucacugccaccauccgccugu	22	1	9	28	1
LL-miR08	c35378.graph_c0	cgguugcuuagcuuguacucu	21	0	12	1	0
LL-miR09	c36638.graph_c0	ugcaccuccuccuccuuuucu	21	56	13	33	244
LL-miR10	CL3742.Contig2_All	gguuugaugaaucugagcauc	21	14	19	55	18
LL-miR11	c53683.graph_c0	uuacgugucccuuaaucugacggg	24	0	3	0	1
LL-miR12	c56352_g1	gcucggguuaacggggaagug	21	9	56	0	0
LL-miR13	c59256_g1	uaugaaguuauauagguuguccgg	24	1	6	0	0
LL-miR14	c79455.graph_c0	uuuacugccaccauccgccugc	22	3	12	5	0
LL-miR15	c94000_g1	aagguaagagaaucaacaagaggu	24	0	8	0	0
LL-miR16	c96112_g1	uaggcaacaaauuagagucucu	22	8	8	33	50
LL-miR17	c99691_g1	uuuguauggucuguugaaauu	21	4	0	0	0
LL-miR18	c109150_g1	caggcggcgaggauggggaug	21	2	5	0	0
LL-miR19	c109175_g1	uugcuuagcuuguacucucgc	21	1	4	4	0
LL-miR20	c110752_g2	ugaaaauguagcacuagcacc	21	1	7	0	0
LL-miR21	c111855_g1	uugagaguagagagccaggug	21	0	12	1	4
LL-miR22	c114504_g1	aaaugaugaaucugagccuc	20	8	0	10	6
LL-miR23	c114692_g4	uagaggcgaugaugaugaaau	21	44	795	75	119
LL-miR24	c117497_g1	ugaagacuuggcaaccgacauc	22	4	20	2	0
LL-miR25	c117720_g1	ucugcccugauaugagcuccag	22	36	0	137	0
LL-miR26	c117786_g3	ucugaauagcaaacccaauuc	21	3	5	1	0
LL-miR27	c166092_g1	aaacgaucgauaaaccucugc	21	0	4	2	0
LL-miR28	c48903.graph_c0	aaugagaagacuagugacaagauu	24	4	73	0	0
LL-miR29	CL711.Contig2_All	uucccuucggcugcaaauagc	21	77	25	47	33
LL-miR30	CL719.Contig1_All	uagaggcgaugaugaugaaau	21	0	2	0	1
LL-miR31	CL1297.Contig2_All	aucuuuggccuggagauagagg	22	0	3	0	0
LL-miR32	c71927_g1	ugugccaugcugugugcgucc	21	2	30	2	0
LL-miR33	CL4047.Contig1_All	ugccgggcuaagauacaaggau	22	1	0	2	1
LL-miR34	HM045458.1	ucuauaugacucucggcaacgg	22	0	1	25	2
LL-miR35	JZ391002	uccaaagucagugaggggagc	21	0	9	0	0
LL-miR36	Unigene13110_All	uucgagugacauauggaaacu	21	1	3	0	0
LL-miR37	Unigene18554_All	ucaaucuuuggccuggagauagag	24	2	10	4	0
LL-miR38	c68386.graph_c0	ugggucuccucucauuccaug	21	9	13	0	0
LL-miR39	Unigene25443_All	uucgagugacauauggaaacu	21	1	3	0	0
LL-miR40	c51021.graph_c0	ucaaagacgaaucugagcaua	21	2	6	0	0
LL-miR41	c56504.graph_c0	ucguaucugugguuugcuccu	21	0	1	2	0
LL-miR42	c59249.graph_c0	ugcaguuugguuuguggugug	21	1	3	0	1
LL-miR43	GW589960	cugucgagcuuccauacuggc	21	0	3	0	0
LL-miR44	JZ391211	uggaucuugaaccaaguguuc	21	0	2	10	0

### Prediction of miRNA targets

Plant miRNAs play important roles in diverse biological processes by cleaving target mRNAs or suppressing the translation of target genes. In order to understand the biological functions of *L. lancifolium* miRNAs, TargetFinder and psRNA Target software was used to predict putative target genes of novel and conserved miRNAs. The results from analysis showed that 366 target genes for 17 conserved miRNA families and 415 target genes for 40 novel miRNAs were predicted (Supplementary Tables S4 and S5). The majority of the target genes for conserved miRNAs were transcriptional factors, and many target genes were conserved between *Lilium* and other plant, such as *squamosa promoter-binding proteins* (*SPL*), *MYB*, *proliferating cell factors* (*PCF*), *auxin response factor* (*ARF*), *DCL1*, *NAC domain transcription factor*, *cup-shaped cotyledon 2*(*CUC2*), *PHB*, *AGO1*, *APETALA*, *ATP sulfurylases*, *growth-regulating factors* (*GRF*), *UBC24* and *blue copper protein*, which were involved in various aspects of plant growth and development. However, some predicted target genes of several conserved miRNAs in *L. lancifolium* were different from those in other plants, including *fasciclin-like arabinogalactan protein* (*FLA*) and *homeobox-leucine zipper protein HOX* (MIR166), *ethylene-responsive transcription factor RAP2-7* (MIR172), *LRR receptor-like serine/threonine-protein kinase* (MIR390), *stellacyanin* and *cucumber peeling cupredoxin* (MIR398). Interestingly many target genes for miR845 were predicted in *L. lancifolium* like *patatin protein*, *phosphatidate cytidylyltransferase*, *dnaJ protein*, and *LRR receptor-like serine/threonine-protein kinase* (Supplementary Table S4). The target genes of novel miRNAs were mainly predicted to be protein coding genes, such as *polyphenol oxidase*, *polycomb group protein FIE2*, *serine/threonine-protein kinase*, *pentatricopeptide repeat-containing protein*, *DRM-type DNA-methyltransferase*, *ATP-dependent DNA helicase*, *F-box protein* and so on (Supplementary Table S5).

### Validation of miRNAs using northen hybridation and stem-loop RT-PCR

**Figure 2:**
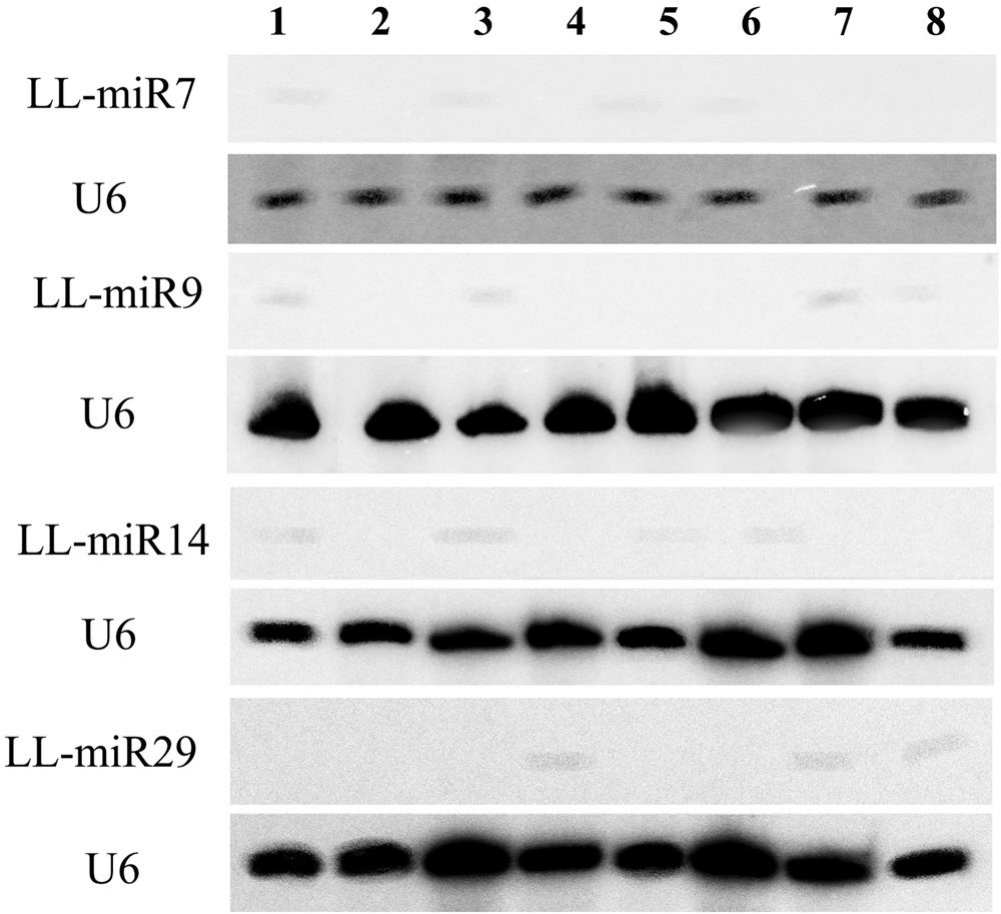
Detection of four novel miRNAs using Northern blotting. Lane 1–4, *L. lancifolium*; Lane 5,6, Brunello; Lane 7,8, White heaven; Lane1,5,7, flower; Lane 4,6,8, leaf; Lane2, bulblet; Lane3 bulb. U6 RNA is used as a loading control. The full-length blots are presented in Supplementary Fig. S3.

**Figure 3:**
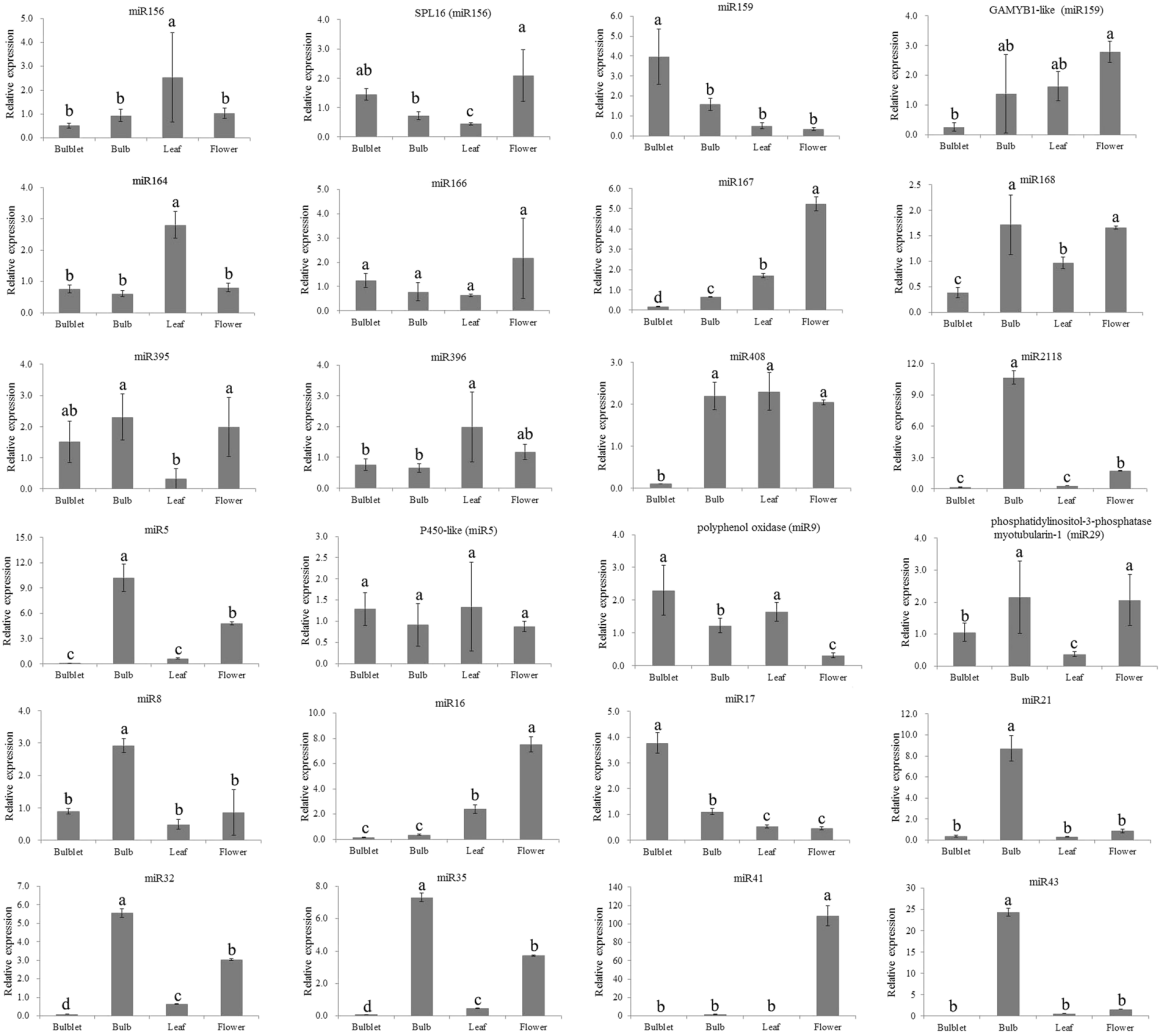
Validation of miRNAs and targets using qRT-PCR. The X axis represents different tissues. The Y axis represents the relative expression level of miRNAs or targets.The amount of expression of miRNAs and targets was normalized to the level of 5.8S rRNA and 18S rRNA, respectively. Different letters indicate significant differences at P < 0.05 according to Duncan’s multiple range tests.

To confirm the credibility of miRNAs from the high-throughput sequencing and bioinformatics analysis, 4 novel miRNAs (LL-miR7, LL-miR9, LL-miR14 and LL-miR29) were randomly selected and their expressions in flower, leaf, bulblet and bulb of *L. lancifolium* were detected using northern blot analysis. All of them were detected in at least one tissue (Fig. 2). Two lily cultivars, ‘Brunello’ and ‘White heaven’, were also used for northen hybridization. All 4 novel miRNAs were detected in ‘Brunello’ or ‘White heaven’. In addition, 19 miRNAs (10 conserved and 9 novel miRNAs) were randomly selected for stem-loop RT-PCR analysis. All of these miRNAs were detected from flower, leaf, bulblet and bulb of *L. lancifolium*. Among them, 7 miRNAs (LL-miR2118, LL-miR5, LL-miR8, LL-miR21, LL-miR32, LL-miR35 and LL-miR43) were found primarily in bulb. Three miRNAs (LL-miR167, LL-miR16 and LL-miR41) were expressed predominantly in flower (Fig. 3). Three miRNAs (LL-miR159, LL-miR156 and LL-miR164) were highly expressed in bulblet or leaf. For the specificity and sensitivity of northern hybridization and stem-loop qRT-PCR^34,35^, the identification of conserved and novel miRNAs in *L. lancifolium* was effective and credible.

### Verification of miRNA targets by degradome sequencing and RT-PCR

In order to validate the targets of conserved and novel miRNAs in *L. lancifolium*, the high-throughput degradome sequencing was used to detect the cleavage site of predicted targets. A total of 683,113,369 raw data were obtained. After removing adaptors, low quality reads and repeat sequences, 14,534,088 clean tags were yielded. A total of 12,194,038 clean tags were mapped perfectly to *Lilium* RNAs. After bioinformatics analysis, 27 and 26 cleavage sites were identified to 6 conserved miRNAs families and 14 novel miRNAs, respectively (Supplementary Tables S4 and S5). According to Addo-Quaye’s analysis method^36^, all targets identified by degradome sequencing could be classified into five categories: twenty-two cleavage sites with category 0, ten cleavage sites with category 1, nine cleavage sites with category 2, five cleavage sites with category 3, seven cleavage sites with category 4. Most cleavage sites (11/12) with category 3 and 4 were from novel miRNA targets. These targets of conserved miRNAs included *SPLs*, *MYB*, *GRFs*, *DCL1*, *pentatricopeptide repeat-containing protein*, *Ras-group-related LRR protein*, *methyltransferase PMT7*, *chorismatemutase 3* and *trifunctional UDP-glucose 4,6-dehydratase* (Supplementary Table S4). Novel miRNAs could cleave *P450-like, polyphenol oxidase*, *auxin response factor 7-like*, *Kunitz-type trypsin inhibitor*, *F-box protein*, *ferredoxin*, *FLS2*, *N-alpha-acetyltransferase 16*, and so on (Supplementary Table S5).

To validate the regulation of target expression by miRNA, five targets for two conserved and three novel miRNAs were investigated using qRT-PCR. Among them, LL-miR9 could be detected in flower and bulb using northern blot. *Polyphenol oxidase*, a predicted target of LL-miR9, has higher expression in bublet and leaf. *Phosphatidylinositol-3-phosphatase myotubularin-1* to potentially be targeted by LL-miR29 has decreased expression in leaf (Fig. 3). The result agreed with the expression of LL-miR29 from northern blot. In addition, LL-miR156 and LL-miR159 also had the opposite expression pattern with their targets (*SPL16* and *GAMYB1-like*).

## Discussion

*Lilium* species belong to genus Liliaceous, known as perennial herbaceous flowering plants growing from bulbs and among the most important cut flowers worldwide. Although several research reports shown that miRNAs play crucial roles in plant growth, development, and response to stress in plants, however, there is no report on *Lilium* miRNAs identification. The high-throughput sequencing is an effective method to screen miRNAs from various plants. Up to date, many miRNAs from ornamental plant, including *Lycoris aurea*, *Nelumbo nucifera*, *Herbaceous peony*, *Cymbidium ensifolium*, *Ginkgo biloba*, *Prunus mume* and rose^21,37–42^, have been identified using this method. To discover miRNAs in *Lilium* and understand their functions, we constructed and sequenced four small RNA libraries from flower, leaf, bulblet and bulb of *L. lancifolium*. The result of this research adds our knowledge to understand about the role of miRNAs from Liliaceae, in which a number of genera are popular cultivated plants with ornamental value.

The study on miRNAs in *Lilium* was limited to their large genome (~36 Gb) compared with other plants in which genome has been sequenced, such as *Arabidopsis*^43^, rice^44^, wheat^45^, Populus^46^. In this study, RNA sequences from *Lilium* in NCBI database, transcriptome data from *Lilium pumilum* and an Asiatic hybrid cultivar ‘Easy Dance’ besides to *L. lancifolium* were also used to predict miRNAs and their targets. As a result a total of 17 conserved miRNA families and 44 novel miRNAs were identified compared with only 9 conserved miRNA families and 17 novel miRNAs using *L. lancifolium* RNA. Therefore, we believe that deep sequencing of different tissues at various developmental stages could be necessary to fully disclose the miRNA function in *Lilium*. And in comparison to other close physiologically plants, the number of miRNAs identified from *L. lancifolium* are less than rice (713), wheat (119), maize (321), *Brachypodium distachyon* (525) which have been well genomic sequenced, but more than those plants without genome sequencing data, like *Elaeis guineensis* (6), *Festuca arundinacea* (15), *Saccharum officinarum* (16) (miRbase database release 21).

The lengths of plant sRNAs usually ranged from 21 nt to 24 nt^47^. Among them, 24 nt sRNA was the most abundant, followed by 21 nt class^13,48,49^. However, our research result shows that 21 nt sRNAs were more abundant in flower and bulb whereas 23 nt sRNA in leaf and bulblet were more abundant than 24 nt sRNA in *L. lancifolium*. It has been reported that the 21 nt sRNA is the most abundant sRNA species in *Populus balsamifer*, Chinese Wild *Vitis pseudoreticulata* and *Pinus cordata*^48,50,51^. Our results from flower and bulb supported the speculation given by Han and colleagues that the major sRNA species were 21 nt in perennial plants^51^. The 23 nt sRNAs could arising from loci dominated by 24 nt siRNAs^52^ and *MIR* genes^53^, but their biological functions are less understood. Therefore, a large number of 23 nt sRNA may play a special role in leaf and bulblet of *L. lancifolium*.

After analyzing miRNAs identified in this study, we found that the 5′ ends of most miRNAs were U (84%for conserved miRNAs and 70.8% for novel miRNAs) in *L. lancifolium*. The result was consistent with previous reports that the U at 5′end of miRNA favored the combination with AGO1^54^. It has been reported that miRNAs had differential accumulation patterns in many plant species, and possessed their own precise regulation processes through the tissue dependent miRNA biogenesis in different plant species^11,55^. In this study, the expression analysis of miRNAs from qRT-PCR and northern blot showed that many conserved and novel miRNAs were tissue biased in *L. lancifolium*. This result suggested that the miRNAs might play very important roles in development of different tissue.

In order to understand the functions of miRNAs in *L. lancifolium*, a large number of targets of conserved and novel miRNAs were predicted in this research. These conserved miRNAs LL-miR156, LL-miR159, LL-miR319, LL-miR160, LL-miR164, LL-miR166, LL-miR172 and LL-miR396 targeted SPLs, MYBs, PCFs, ARFs, NACs, HD-ZIP III, AP2s and GRFs, respectively. Our result is in agreement with previous research that the targets of conserved miRNAs in plant are mainly transcription factors^56^. These conserved miRNAs and their transcription factor targets in lily might have similar functions on plant growth and development as those in other plant species. However, the LL-miR398 has been predicted to target type I blue copper proteins rather than reported CSD, CoX5b-1 and CCS1, which were involved in responses to environmental stresses^57–59^. In this study, 27 targets of LL-miR156, LL-miR159, LL-miR162, LL-miR390, LL-miR396 and LL-miR2118 have been validated by degradome sequencing. The result from qRT-PCR analysis showed that LL-miR156 and LL-miR159 had the opposite expression pattern with their targets.

The novel miRNAs were mainly predicted to be protein coding genes. Among 415 predicted targets, 26 were validated by degradome sequencing. Although some cleavage sites for predicted targets, which were matched very well with novel miRNAs, were not detected using degradome sequencing, it was possible that novel miRNAs regulated them at the level of translation. The novel LL-miR09 and LL-miR35 were predicted to target polyphenol oxidase, and the cleavage site of LL-miR09 has been identified by degradome sequencing. In *Populus* and *Salvia miltiorrhiza*, miR1444 and Smi-miR12112 have been reported to regulate a subset of polyphenol oxidases, which have important roles in plant development and response to biotic and abiotic stresses^60–62^. LRR receptor-like serine/threonine-protein kinase FLS2 and ERECTA were predicted as targets of LL-miR21 and miR35, respectively, and the cleavage site of FLS2 has been detected. The FLS2 has been reported to perceive the bacterial elicitor flagellin in *Arabidopsis*^63^. The ERECTA was involved in the thermo tolerance, stomatal development, plant architecture in *Arabidopsis*^64^. The serine/threonine-protein kinase ACR4 targeted by LL-miR25 plays important roles in cell division and differentiation in *Arabidopsis*^65^. The polycomb group protein FIE2, which prevents fertilization-independent seed development in *Arabidopsis*^66^, is a potential LL-miR08 target. The pentatricopeptide repeat-containing protein, which was potentially targeted by LL-miR18, LL-miR43 and LL-miR44, could affect chloroplast development in *Arabidopsis*^67^. DRM-type DNA-methyltransferase involved in RNA-directed DNA methylation in *Arabidopsis*, was predicted as target of miR20^68^. ATP-dependent DNA helicase DDM1 participated in UV-B induced and oxidative DNA damage repair in *Arabidopsis*^69^, was potentially targeted by LL-miR36. F-box protein potentially targeted by LL-miR26, LL-miR38 and LL-miR44, has been reported to mediate bouquet formation to promote homologous pairing, synapsis, and recombination in rice meiosis^70^. It is very interesting that LL-miR07 and LL-miR14 owed highly similar mature miRNA and precursor sequences. Therefore, we speculated that LL-miR07 and LL-miR14 might be derived from the same ancestor. Both of them were predicted to target ARF7-like, which regulates lateral root formation, differential growth of hypocotyls in *Arabidopsis* and fruit set in tomato^71,72^. Since these novel miRNAs were predicted to participate in plant growth, development, biotic and abiotic stress responses, and signal transduction and so on, further studies are recommended to understand the functions of novel miRNAs in *Lilium species*.

## Methods

### Plant material and RNA preparation

*L. lancifolium* was selected as the experiment material, and grown in the greenhouse of Beijing University of Agriculture, Beijing, China. Flowers, leaves, bulblets and bulbs of *L. lancifolium* were collected and immediately frozen in liquid nitrogen. The frozen samples were then stored at −80 °C for future analysis. The TRIzol® reagent (Invitrogen, USA) was used to extract total RNA from four samples according to the manufacturer’s protocol. Finally, the integrity of total RNA was confirmed using 1% agarose gel electrophoresis and Agilent 2100 Bio analyzer (Agilent Technologies, USA).

### Small RNA library construction and sequencing

Small RNA libraries of flowers, leaves, bulblets and bulbs were constructed using previously described methods^73^. Briefly, small RNAs fragments of 10–30 nt were purified from a 15% denaturing polyacrylamide gel and then ligated with 5′ and 3′ adapters. After being reverse-transcribed by Superscript II reverse transcriptase (Invitrogen) and amplified by PCR, about 20 µg products from each sample were sequenced using Illumina HiSeq. 2500 sequencing platform (Illumina Inc.; San Diego, CA, USA) at the Biomarker Technologies (Beijing, China).

### Prediction of conserved and novel miRNA

After removal of chip adaptor sequences, low quantity reads and contaminations, the clean 18–30 nt small RNAs were mapped to GenBank (http://www.ncbi.nlm.nih.gov/) and Rfam (version 10.1) database (http://rfam.sanger.ac.uk) with a cut-off value of 0.01, and rRNA, tRNA, snRNA, snoRNA were removed to produce filtered small RNAs. All available *Lilium* RNA sequences were collected to predict miRNAs and their targets, including unigenes from transcriptome sequencing of *L. lancifolium* (SRA632698), *Lilium pumilum* (SRA633315), Asiatic hybrid lily cultivars ‘Easy Dance’ (SRA538278), and 3902 ESTs (*Lilium formosanum*, *Lilium longiflorum*, *Lilium regale*, Lilium hybrid division VII, and *Lilium davidii* var. willmottiae) downloaded from NCBI. All filtered small RNA, which were aligned against miRbase database (release 21) (http://www.mirbase.org/) with no more than two mismatches, were aligned against *Lilium* RNA sequences. Their flanking sequences were fold with mfold soft^74^. The filtered small RNA in perfect stem-loop structure was considered as conserved miRNAs. The miRDeep2 with modified parameter was used to identify novel miRNAs and check the secondary structures of putative pre-miRNAs^75^. The minimum free energy index (MFEI) was calculated using the equation: MFEI = AMFE/(G + C)%. The adjusted MFE (AMFE) represented the MFE of 100 nucleotides. It was calculated using (MFE/length of RNA sequence) × 100^76^.

### Target prediction of conserved and novel miRNA

*Lilium* RNA sequences above for miRNA prediction were also used for target prediction. TargetFinder and psRNA Target were applied to predict the putative targets of conserved and novel miRNA^77,78^.

### Quantitative real-time PCR analysis of miRNAs and targets

The stem-loop RT-PCR was used to validate the miRNAs from deep sequencing and to analyze their expression patterns. Total RNA of flower, leaf, bulblet, and bulb were extracted using Trizol reagent (Invitrogen) according to the manufacturer’s instruction. Then, total RNA was reverse-transcribed to cDNA using stem-loop RT primer by the PrimeScript RT reagent Kit (TaKaRa, Dalian, China) according to the manufacturer’s protocol. All primers for stem-loop RT-RCR were designed according to the Chen’s report^35^ and listed in Supplementary Table S6. The qRT-PCR reactions were performed using the SYBR Premix Ex Taq II solution (TaKaRa, Dalian, China) as the following condition: 95 °C for 5 minutes, then 40 cycles of denaturation at 95 °C for 15 s, 60 °C for 30 s, and 72 °C for 30 s. For the qRT-PCR analysis of the miRNA targets, total RNA was used for synthesizing reverse transcripts using PrimeScript RT reagent Kit (Takara, Dalian, China) according to the manufacturer’s instructions. Specific primer pairs for miRNA targets were designed to amplify cDNA (Supplementary Table S6). qPCR was performed using SYBR Premix Ex Taq II (TaKaRa, Dalian, China) under the following conditions: 40 cycles at 95 °C for 15 s, 60 °C for 30 s, and 72 °C for 30 s. The qRT-PCR reactions were performed on the BIO-RAD iQ5 (Applied Biosystems, Foster City, CA). Each sample was processed in triplicate, and the relative expression were calculated using 2^−ΔΔCT^ ^79^. The 5.8S rRNA^80^ and 18S rRNA were used as references to normalize the expression level of miRNAs and their targets. The data were statistically analyzed using SAS Version 9.0 software (SAS Institute, Cary, NC, USA) using Duncan’s multiple range test at the P < 0.05 level of significance.

### Degradome library construction and target identification

To investigate the potential targets of conserved and novel miRNAs, a degradome library was constructed using mixture of mRNA from flowers, leaves, bulblets and bulbs of *L. lancifolium* according to the parallel analysis of the RNA ends protocol^81^, and sequenced using Illumina HiSeqTM 2500 sequencing platform (Illumina Inc.; San Diego, CA, USA) at the Beijing Genomics Institute (BGI) (Shenzhen, China). A Public software package, CleaveLand3.0 was used for analyzing sequencing data. All the putative target genes were used as queries to align against *Lilium* RNA sequences. The true miRNA cleavage sites from background noise were identified using a target plot^82^.

### Northern blot analysis

Total RNA was isolated from plant tissues using TRIzol reagent (Invitrogen, USA) according to the manufacturer’s instructions. For Northern blots, 40 µg of total RNA was separated by electrophoresis on 17% polyacrylamide gel and electrically transferred to nylon N+ membrane. Blots were hybridized with [ϒ-^32^P] ATP-labeled oligonucleotide probe. Hybridization signal intensity was measured using a PhosphorImager (GE Healthcare). Sequences of the oligonucleotide probes are listed in Supplementary Table S6.

### Data availability

The raw data (Accession Number: SRA633909) in the study can be obtained from SRA database.

## Electronic supplementary material

Supplementary Table S1

Supplementary Figures S1 and S2

Supplementary Table S2 

Supplementary Table S3 

Supplementary Table S4 

Supplementary Table S5 

Supplementary Table S6 

Supplementary Figure S3
