# Supplementary material for: Characterization of Conserved and Novel microRNAs in Lilium lancifolium Thunb. by High-Throughput Sequencing
Source: Sci Rep. 2018 Feb 13;8:2880. doi: 10.1038/s41598-018-21193-4 (PMC5811567; doi:10.1038/s41598-018-21193-4)
Supplement: Supplementary file 1 — Supplementary Table S1 [file 41598_2018_21193_MOESM1_ESM.pdf]

Characterization of Conserved and Novel microRNAs in *Lilium lancifolium* Thunb.by High-Throughput Sequencing

Xiangfeng He [1,2,3], Awraris Getachew Shenkute[4], Wenhe Wang [1,2,3,*] , ShufaXu[4,*]

[1] Beijing Engineering Research Center of Rural Landscape Planning and Design, College of Landscape Architecture, Beijing University of Agriculture, Beijing 102206, China

[2] Beijing Collaborative Innovation Center for Eco-Environmental Improvement with Forestry and Fruit Trees, Beijing 102206, China

[3] Beijing Laboratory of Urban and Rural Ecological Environment, Beijing 100083, China

[4] Key Laboratory of Pollinating Insect Biology, Ministry of Agriculture, Institute of Apicultural Research, Chinese Academy of Agricultural Sciences, Beijing 100093, China

E-mail addresses of the authors:

Xiangfeng He: hxf791230@163.com

AwrarisGetachewShenkute:awraris2007@yahoo.com

Wenhe Wang: wwhals@163.com

ShufaXu: xushufa@caas.cn

*Corresponding authors: Dr. Wenhe Wang and Dr. ShufaXu

Supplementary Table S1 RNA quantity and quality measured by Bioanalyzer 2100

Sample	RIN	28S/18S	RNA concentration (ng/μL)
Bulb	8.6	2.0	173
Flower	9.2	1.8	301
Leaf	8.5	1.9	407
Bulblet	8.4	1.5	115
