# Supplementary material for: Characterization of Conserved and Novel microRNAs in Lilium lancifolium Thunb. by High-Throughput Sequencing
Source: Sci Rep. 2018 Feb 13;8:2880. doi: 10.1038/s41598-018-21193-4 (PMC5811567; doi:10.1038/s41598-018-21193-4)

Characterization of Conserved and Novel microRNAs in *Lilium lancifolium* Thunb.by High-Throughput Sequencing

Xiangfeng He [1,2,3], Awraris Getachew Shenkute[4], Wenhe Wang [1,2,3,*] , ShufaXu[4,*]

[1] Beijing Engineering Research Center of Rural Landscape Planning and Design, College of Landscape Architecture, Beijing University of Agriculture, Beijing 102206, China

[2] Beijing Collaborative Innovation Center for Eco-Environmental Improvement with Forestry and Fruit Trees, Beijing 102206, China

[3] Beijing Laboratory of Urban and Rural Ecological Environment, Beijing 100083, China

[4] Key Laboratory of Pollinating Insect Biology, Ministry of Agriculture, Institute of Apicultural Research, Chinese Academy of Agricultural Sciences, Beijing 100093, China

E-mail addresses of the authors:

Xiangfeng He: hxf791230@163.com

AwrarisGetachewShenkute:awraris2007@yahoo.com

Wenhe Wang: wwhals@163.com

ShufaXu: xushufa@caas.cn

*Corresponding authors: Dr. Wenhe Wang and Dr. ShufaXu

Supplementary Figure S1 The stem-loop structures of conserved *L.lancifolium* miRNA precursors. (The mature miRNA in red; the miRNA* from small RNA sequencing in purple; the miRNA* from prediction in skyblue)

LL-miR156a (c45184.graph_c0)

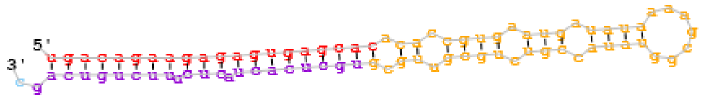

LL-miR156a (c51027_g1)

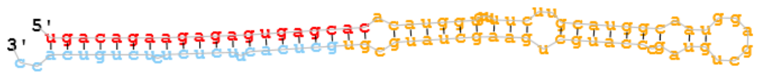

LL-miR156a (c103364_g2)

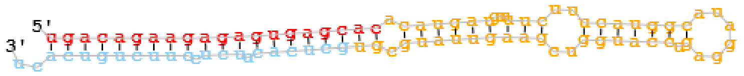

LL-miR156a (c205656_g1)

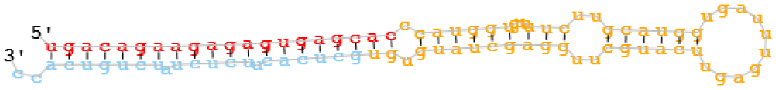

LL-miR156i (c45184.graph_c0)

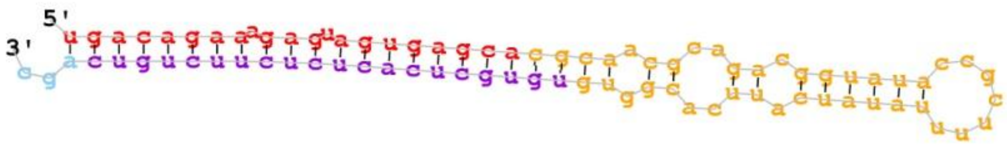

LL-miR159a (CL2574.Contig1_All)

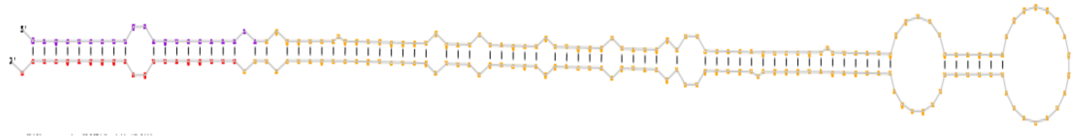

LL-miR159a (CL2574.Contig4_All)

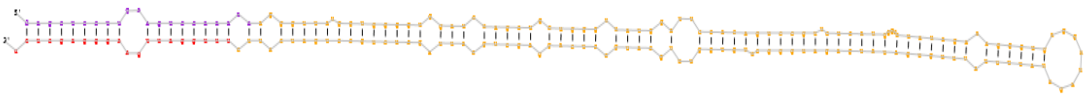

LL-miR159b (c117583_g1)

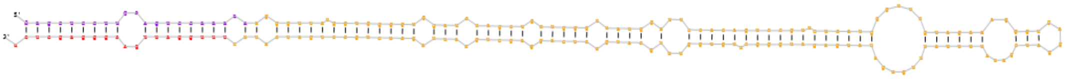

LL-miR159b (c44060.graph_c0)

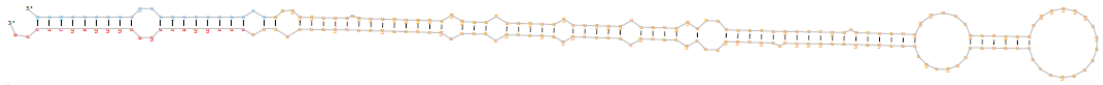

LL-miR319a (c120927_g2)

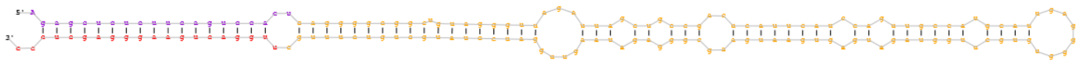

LL-miR160a (c19797.graph_c0)

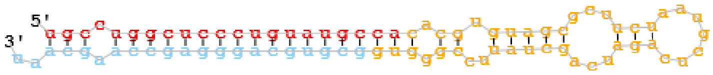

LL-miR160a (c50045_g1)

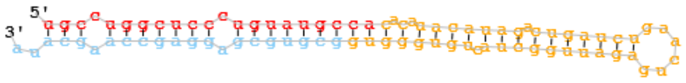

LL-miR160f (c99102_g1)

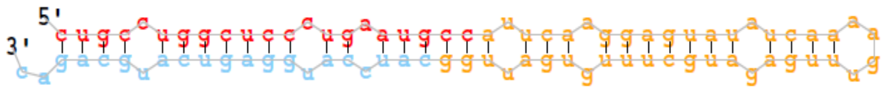

LL-miR162a (Unigene26825_All)

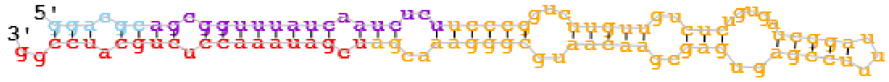

LL-miR164a (c96196_g1)

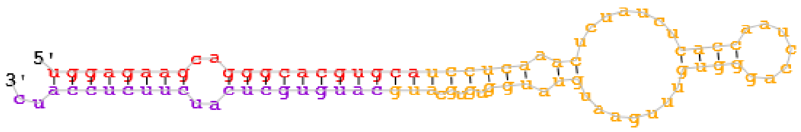

LL-miR164a (c30311.graph_c0)

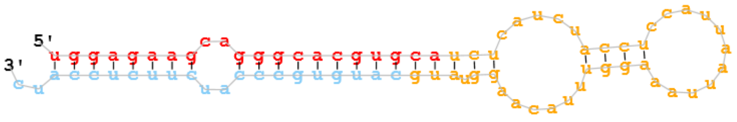

LL-miR166f (Unigene32510_All)

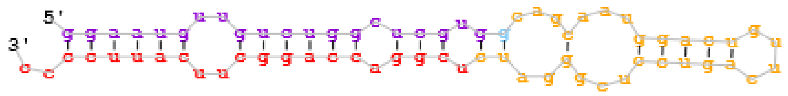

LL-miR166f (c39808.graph_c0)

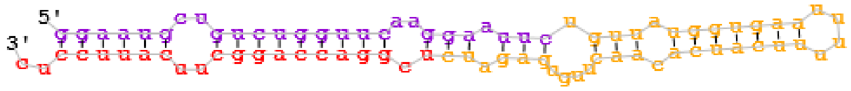

LL-miR166f (c121414_g2)

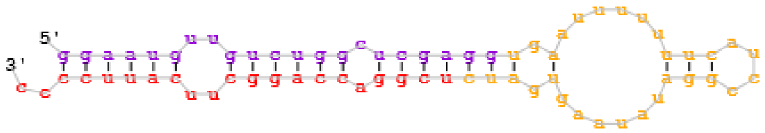

LL-miR166g (c106540_g1)

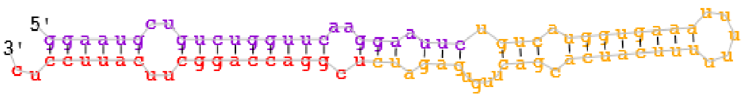

LL-miR167a (c105919_g1)

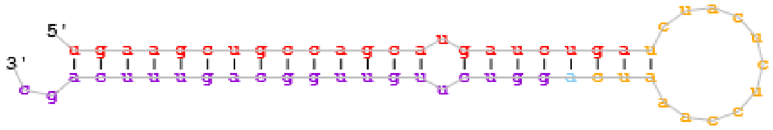

LL-miR168a (c71740.graph_c0)

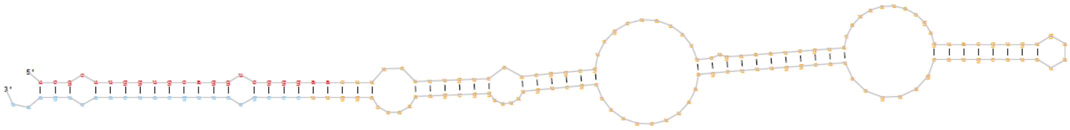

LL-miR168a (c24174.graph_c0)

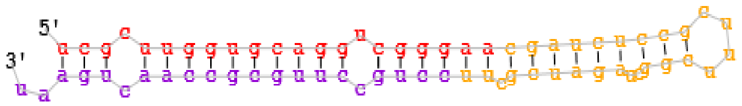

LL-miR168a (c105404_g1)

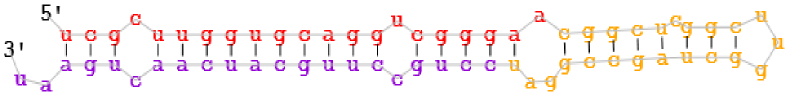

LL-miR172a (c79803.graph_c0)

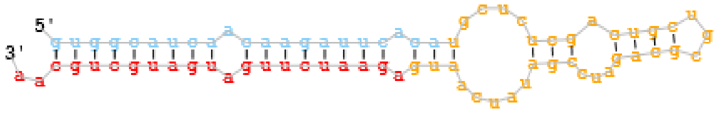

LL-miR390a (c7928.graph_c0)

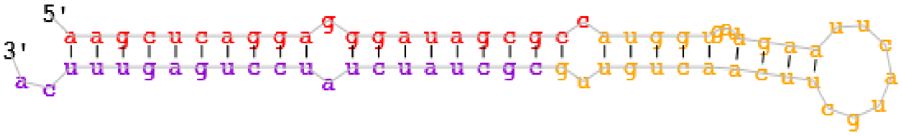

LL-miR395a (c12817.graph_c0)

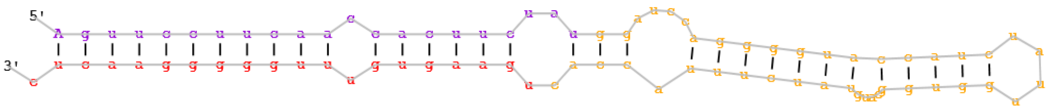

LL-miR395k (c5100.graph_c0)

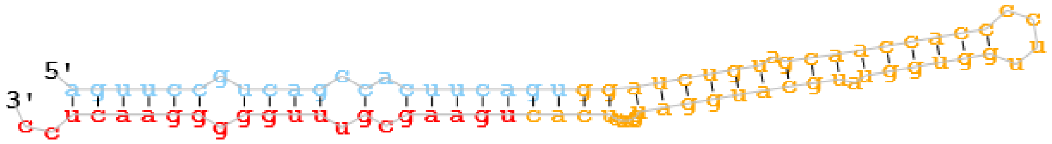

LL-miR396a (c103053_g1)

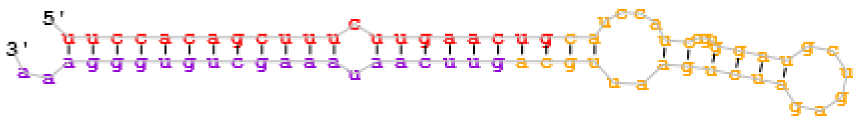

LL-miR396a (Unigene25901_All)

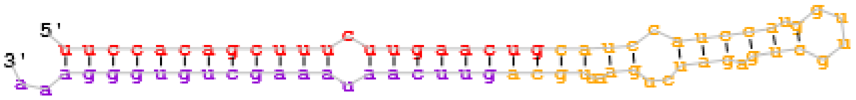

LL-miR396f (c30849_g1)

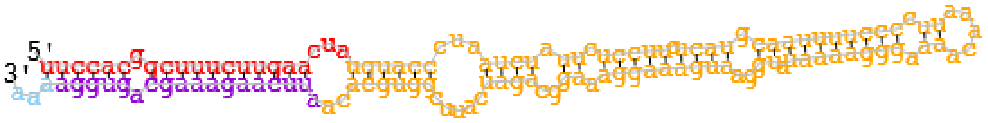

LL-miR398b (c221661_g1)

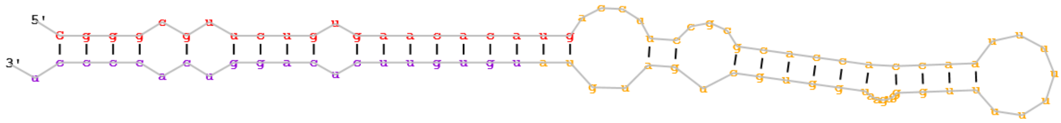

LL-miR398b (c35683_g1)

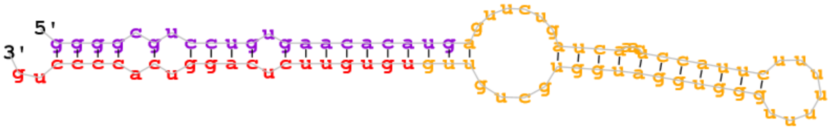

LL-miR399a (c191701_g1)

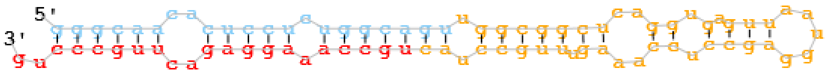

LL-miR408b (c121837_g2)

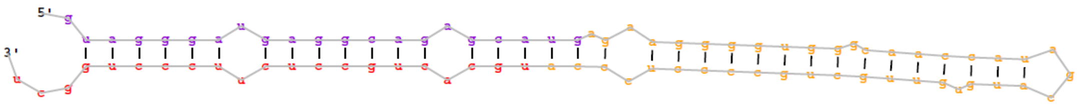

LL-miR845 (c54746.graph_c0)

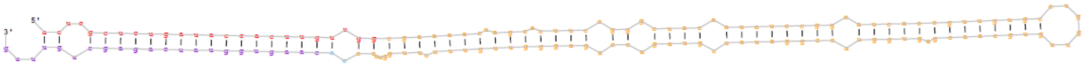

LL-miR2118e (c28582_g1)

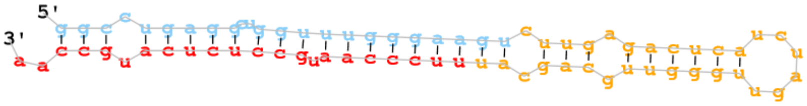

LL-miR2118a (Unigene6944_All)

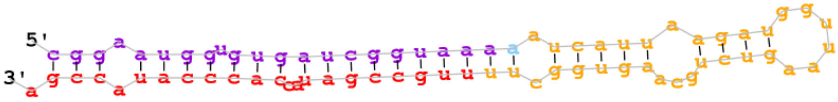

Supplementary Figure S2 The stem-loop structures of novel *L.lancifolium* miRNA precursors. (The mature miRNA in red; the miRNA* from small RNA sequencing in purple; the miRNA* from prediction in skyblue)

LL-miR01 (c33038.graph_c0)

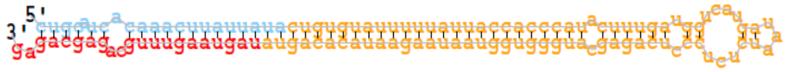

LL-miR02 (c1069.graph_c1)

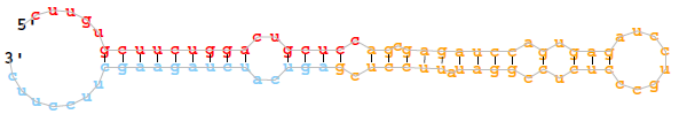

LL-miR03 (c15590.graph_c0)

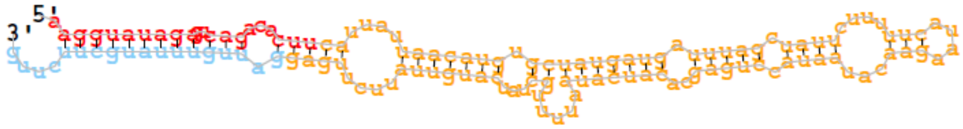

LL-miR04 (c25608.graph_c0)

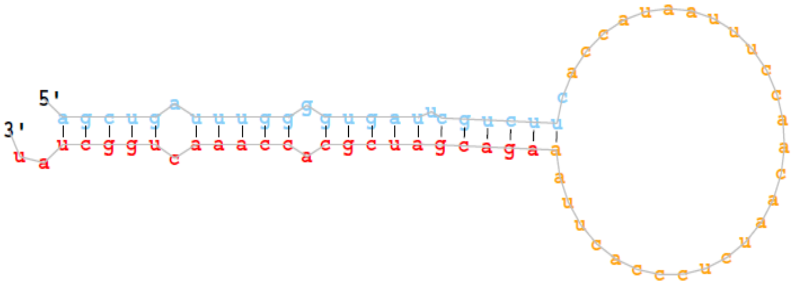

LL-miR05 (c28639.graph_c0)

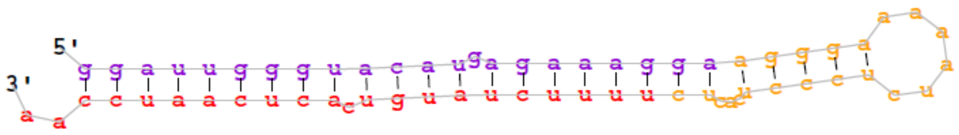

LL-miR06 (c30876.graph_c0)

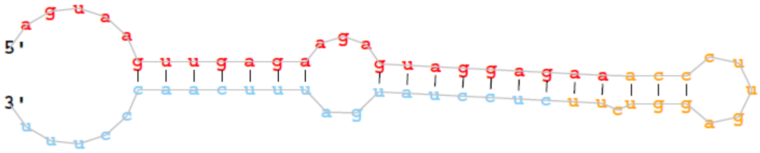

LL-miR07 (c31471.graph_c0)

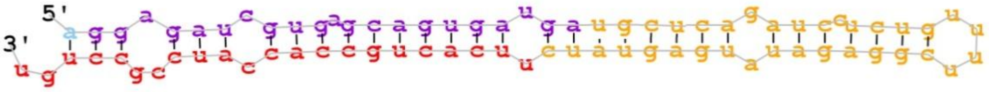

LL-miR08 (c35378.graph_c0)

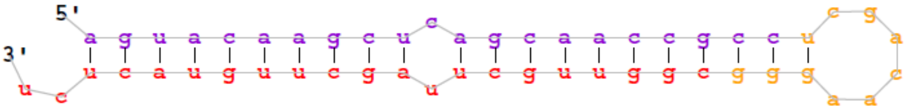

LL-miR09 (c36638.graph_c0)

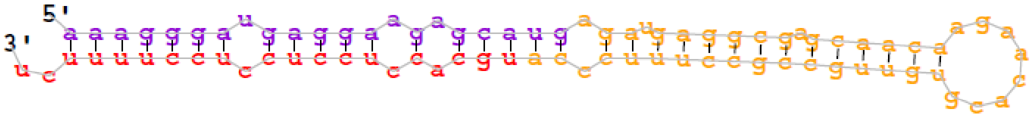

LL-miR10 (CL3742.Contig2_All)

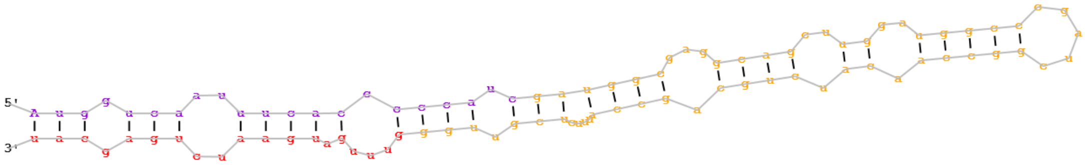

LL-miR11(c53683.graph_c0)

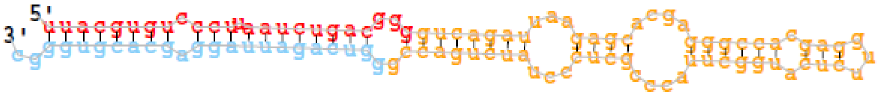

LL-miR12 (c56352_g1)

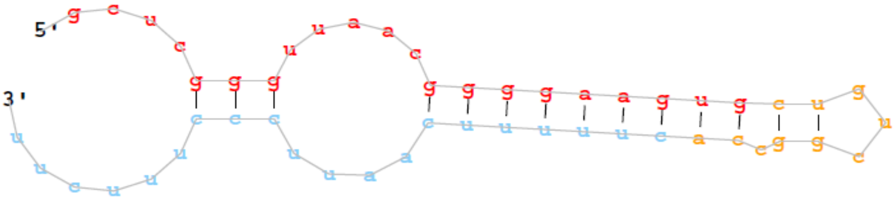

LL-miR13 (c59256_g1)

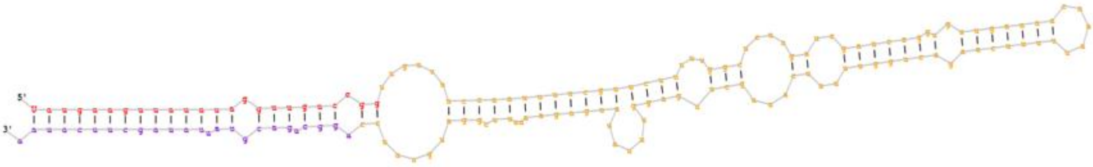

LL-miR14 (c79455.graph_c0)

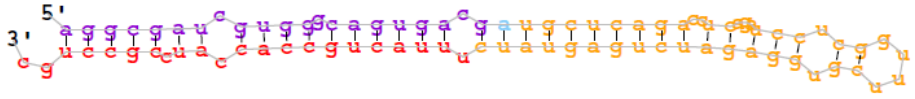

LL-miR15 (c94000_g1)

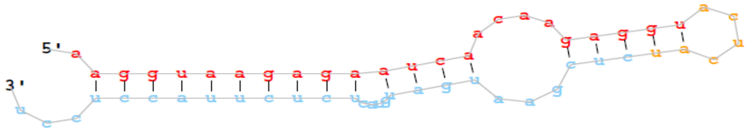

LL-miR16 (c96112_g1)

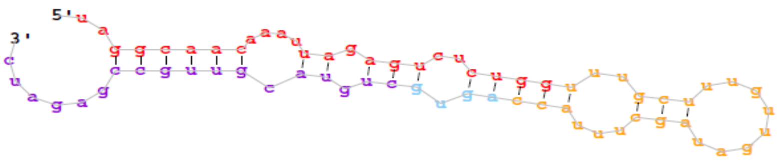

LL-miR17 (c99691_g1)

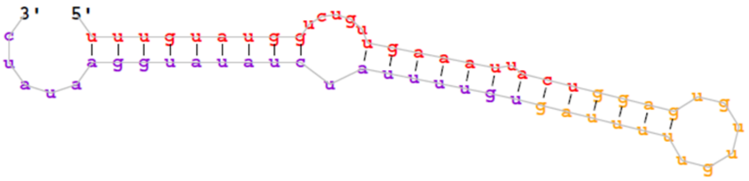

LL-miR18 (c109150_g1)

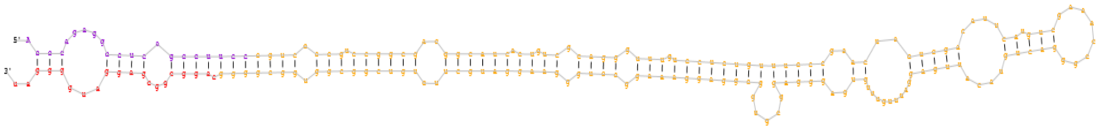

LL-miR19 (c109175_g1)

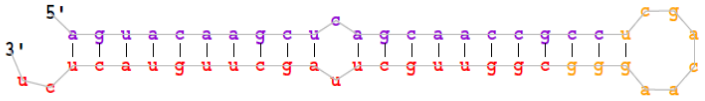

LL-miR20 (c110752_g2)

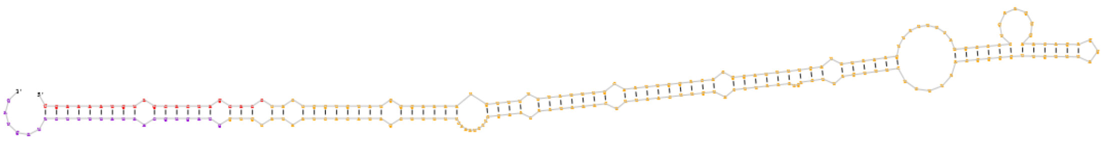

LL-miR21 (c111855_g1)

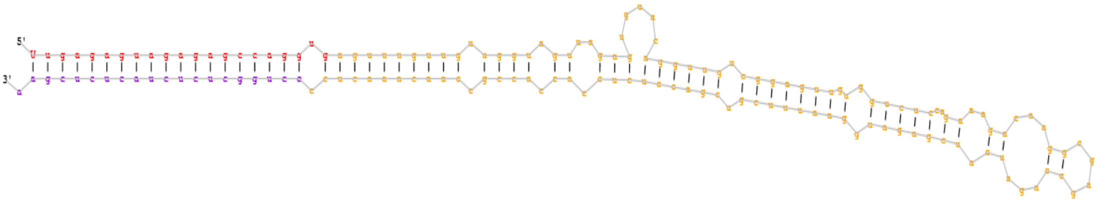

LL-miR22 (c114504_g1)

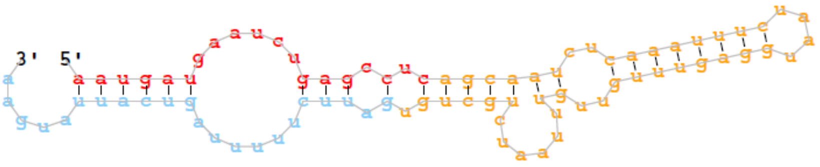

LL-miR23 (c114692_g4)

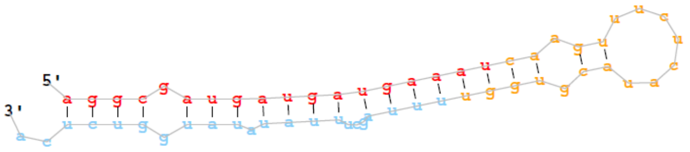

LL-miR24 (c117497_g1)

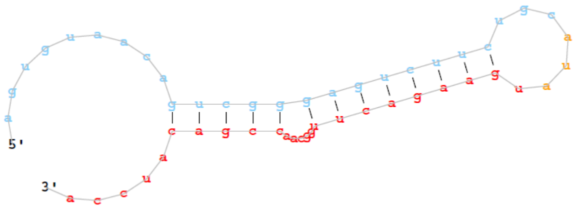

LL-miR25 (c117720_g1)

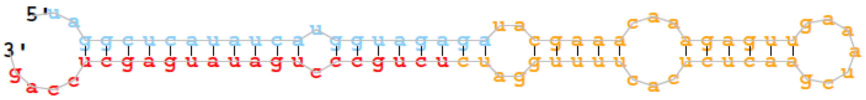

LL-miR26 (c117786_g3)

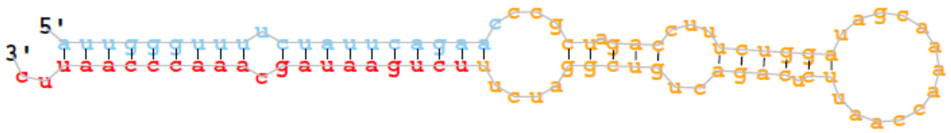

LL-miR27 (c166092_g)

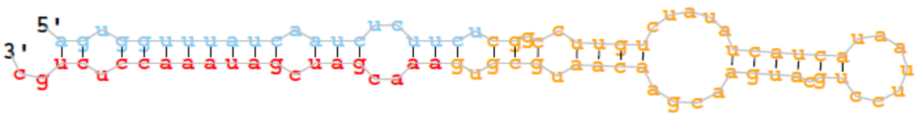

LL-miR28 (c48903.graph_c0)

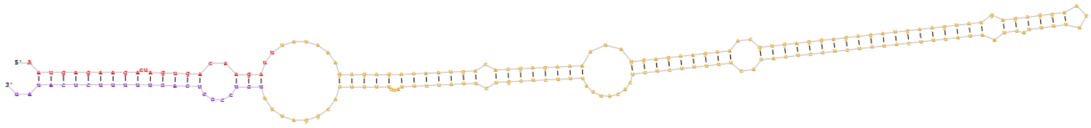

LL-miR29 (CL711.Contig2_All)

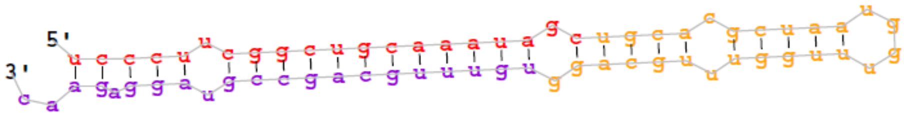

LL-miR30 (CL719.Contig1_All)

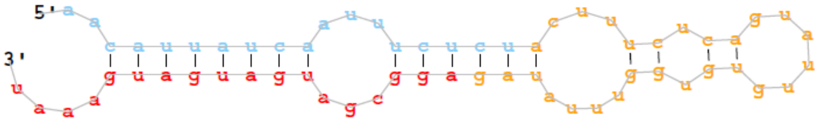

LL-miR31 (CL1297.Contig2_All)

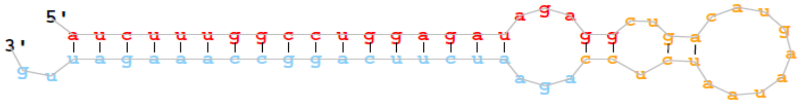

LL-miR32 (c71927_g1)

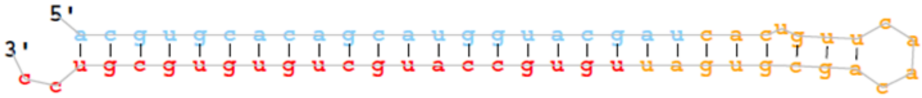

LL-miR33 (CL4047.Contig1_All)

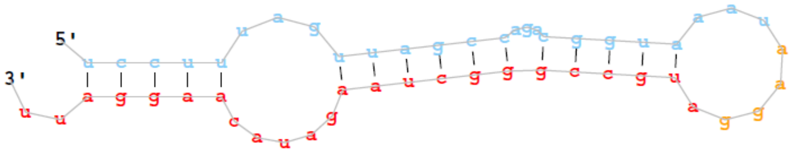

LL-miR34 (HM045458.1)

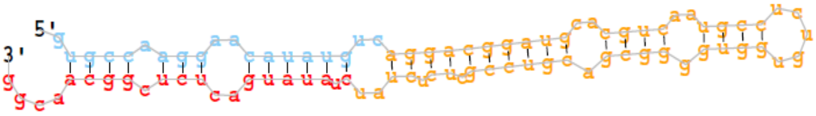

LL-miR35 (JZ391002)

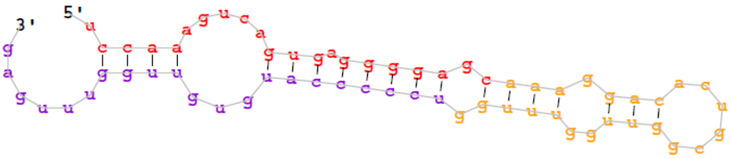

LL-miR36 (Unigene13110_All)

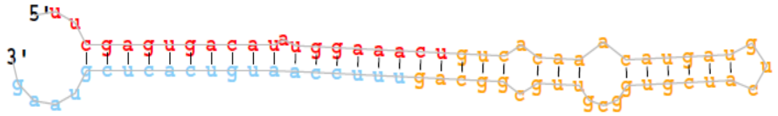

LL-miR37 (Unigene18554_All)

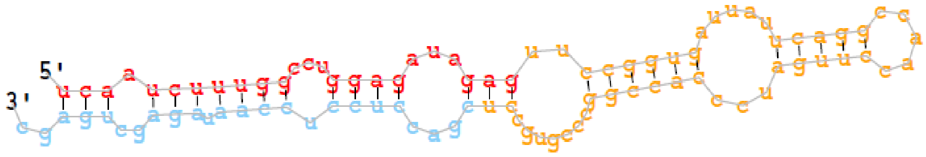

LL-miR38 (c68386.graph_c0)

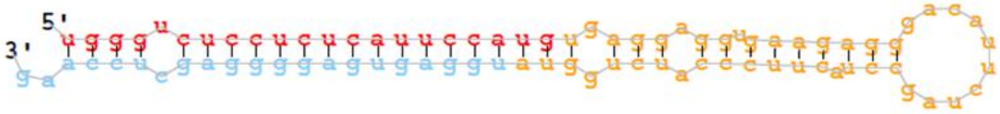

LL-miR39 (Unigene25443_All)

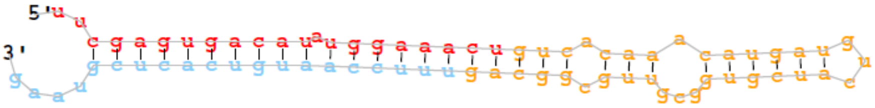

LL-miR40 (c51021.graph_c0)

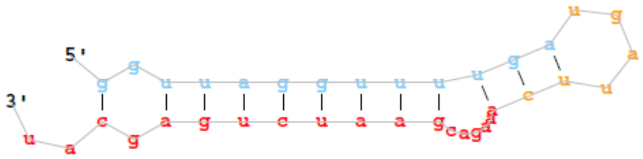

LL-miR41 (c56504.graph_c0)

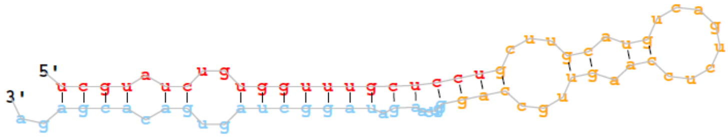

LL-miR42 (c59249.graph_c0)

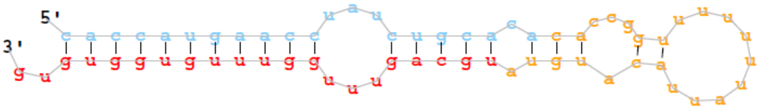

LL-miR43 (GW589960)

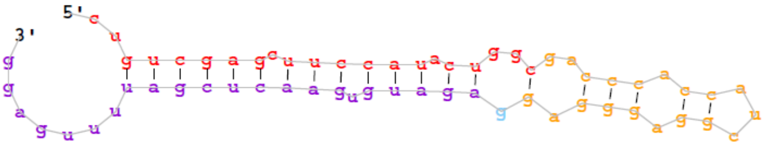

LL-miR44 (JZ391211)

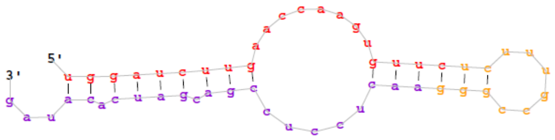

Supplement: Supplementary file 2 — Supplementary Figures S1 and S2 [file 41598_2018_21193_MOESM2_ESM.pdf]
